# The first mainland European Mesozoic click-beetle (Coleoptera: Elateridae) revealed by X-ray micro-computed tomography scanning of an Upper Cretaceous amber from Hungary

**DOI:** 10.1038/s41598-021-03573-5

**Published:** 2022-01-07

**Authors:** Márton Szabó, Robin Kundrata, Johana Hoffmannova, Tamás Németh, Emese Bodor, Imre Szenti, Alexander S. Prosvirov, Ákos Kukovecz, Attila Ősi

**Affiliations:** 1 Department of Paleontology and Geology, Hungarian Natural History Museum, Ludovika tér 2, Budapest, 1083 Hungary; 2 Department of Palaeontology, Institute of Geography and Earth Sciences, ELTE Eötvös Loránd University, Pázmány Péter sétány 1/C, Budapest, 1117 Hungary; 3 Department of Zoology, Faculty of Science, Palacky University, 17. listopadu 50, 771 46 Olomouc, Czech Republic; 4 Department of Zoology and Ecology, Hungarian University of Agriculture and Life Science, 1. Páter K. str., 2100 Gödöllő, Hungary; 5 Institute for Geological and Geochemical Research, Research Centre for Astronomy and Earth Sciences, Eötvös Loránd Research Network, 1112 Budaörsi Street 45, Budapest, Hungary; 6 Department of Applied and Environmental Chemistry, Interdisciplinary Centre of Excellence, University of Szeged, Rerrich Béla tér 1., 6720 Szeged, Hungary; 7 Department of Entomology, Faculty of Biology, Moscow State University, Leninskie gory 1/12, Moscow, Russia 119234

**Keywords:** Zoology, Entomology, Evolution, Palaeontology, Taxonomy

## Abstract

Fossil bioinclusions in amber are invaluable source of information on the past evolution and diversity of various organisms, as well as on the paleoecosystems in general. The click-beetles, Elateridae, which originated and greatly diversified during the Mesozoic, are mostly known from the adpression-like fossils, and their diversity in the Cretaceous ambers is only poorly documented. In this study, we describe a new click-beetle based on an incomplete inclusion in ajkaite, an Upper Cretaceous (Santonian) amber from the Ajka Coal Formation from Hungary. We used X-ray micro-computed tomography scanning to reconstruct its morphology because it is deposited in an opaque piece of amber. Our results suggest that the newly described *Ajkaelater merkli* gen. et sp. nov. belongs to subfamily Elaterinae. It represents the first Mesozoic beetle reported from Hungary, and the first Mesozoic Elateridae formally described from mainland Europe. Our discovery supports an Eurasian distribution and diversification of Elaterinae already in the Cretaceous. The paleoenvironment of the Ajka Coal Formation agrees well with the presumed habitat preference of the new fossil taxon. The discovery of a presumably saproxylic click-beetle shed further light on the yet poorly known paleoecosystem of the Santonian present-day western Hungary.

## Introduction

Fossils play an important role in our understanding of past processes including the origin, evolution and diversification of beetle lineages^1,2^. Among fossils, amber inclusions are especially valuable for scientists because they allow to observe the morphological features in much better detail than e.g., compression fossils^3,4^. Mesozoic amber outcrops are known from various places on the planet^5,6^ but only a few of them dated to the Cretaceous include fossil insects. The most important Cretaceous amber sites are located in Lebanon, Israel, Jordan, France, England, Spain, USA, Canada, Russia and Myanmar^5–15^. The Burmese, Lebanese, Spanish and French sites are particularly interesting due to their great abundance and diversity of insects^5,14–16^. While some amber sites produce many bioinclusions, with numerous new insect taxa being described on a regular basis, some other amber sites are rather poor in the matter of described diversity. For example, bioinclusions have been found only in a fraction of amber sites in France^15,17^, most probably due to a high proportion of the opaque amber stones, in which the inclusions can be detected only using X-ray radiographic and tomographic imaging techniques^15^.

**Figure 1:**
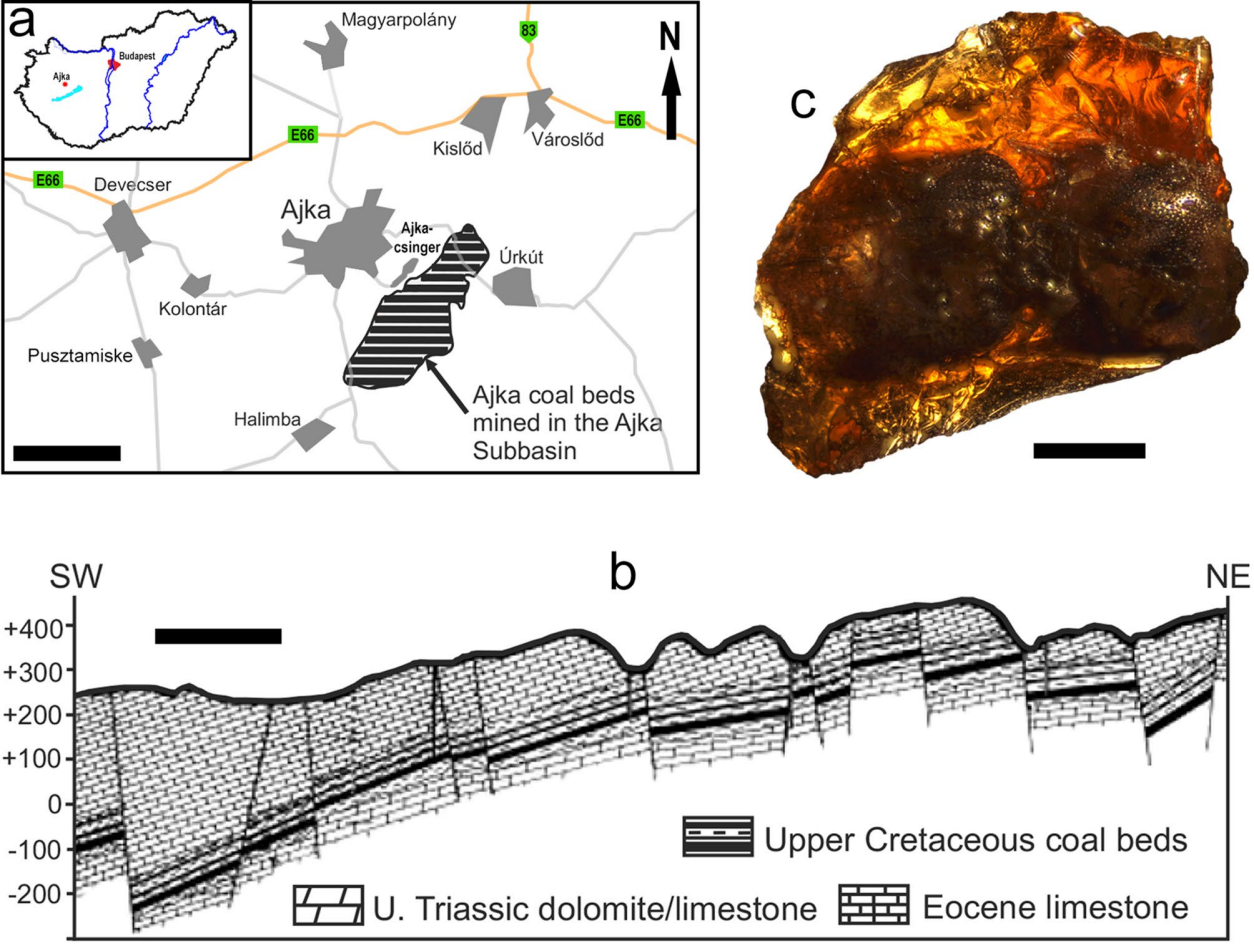
Ajka location and geology, and the here examined amber inclusion. (**a**) Location of Ajka in western Hungary, with the location of the Ajka coal beds. (**b**) Simplified geological section of the Ajka Subbasin. (**c**) Ajkaite specimen containing the holotype of *Ajkaelater merkli* gen. et sp. nov., under polarized light microscope. Scale bars: (**a**) 3 km; (**b**) 1 km; (**c**) 1 mm.

Upper Cretaceous amber-bearing sedimentary units in Hungary include the alluvial floodplain deposits of the Csehbánya Formation nearby Iharkút and the Ajka Coal Formation (Ajkacsinger) southeast from the city Ajka in the Bakony Mountains in southwestern Hungary (Fig. 1), both being Santonian in age. Despite more than a thousand of amber pieces known from the former locality, no inclusions have been found in these dominantly small (up to 3 mm), drop-like pieces so far. On the other hand, the unique type of amber from the brown coal beds of Ajka, so-called ajkaite^18^, contains many bioinclusions.

The Ajka Coal Formation, being at some places over 100 m thick^19,20^, comprises an alternation of coal beds, carbonaceous to argillaceous pelitic sediments with interbedded molluscan lumachelles, marls, and sandstone beds representing a lacustrine-palustrine sequence^21^. It has a well documented fossil flora^22–26^, and fauna of mollusks^27,28^ and vertebrates^29^. Despite a relatively large number of available amber stones from the Ajka deposits, the record of the ajkaite inclusions is still rather scarce. Short mentions on the inclusions in ajkaite are known since the middle of the twentieth century^30,31^. Regarding the arthropods, apart from an officially unpublished MSc thesis^32^, there is only one detailed study by Borkent^33^ describing two species of ceratopogonid flies. Obviously, many other ajkaite arthropods belonging to Arachnida, Diptera, Hymenoptera and Coleoptera are waiting to be formally described^34^.

The Elateridae, commonly known as click beetles, are the largest and most diverse family in the superfamily Elateroidea. Adult individuals can be usually recognized by their elongate, narrow body, structure of basal antennomeres, pro-mesothoracic clicking mechanism, acute posterior angles of pronotum, and five abdominal ventrites, of which four are connate and the last one is free^35–37^. Their most characteristic feature is their ability to jump into the air by rapidly sliding their prosternal process into their mesosternal cavity, with a typical clicking sound, giving their common name^35,37^. The family currently includes more than 11,000 described species worldwide^37,38^ and the fossil record includes 261 species in 99 genera^39,40^. Although they are quite common in various amber deposits worldwide, only a few of them have been described so far^40^. Regarding the Cretaceous click-beetles in amber, only three species have been described to date, all from the Burmese amber^41,42^.

In this study, we describe a new click-beetle inclusion in ajkaite, which represents the first Mesozoic beetle reported from Hungary as well as the first Mesozoic Elateridae described from the mainland Europe. Since this incomplete specimen is deposited in a non-transparent piece of amber, we had to use X-ray micro-computed tomography scanning to reveal its morphology. We discuss the probable ecology of a new click-beetle in connection with the Ajka Coal paleoenvironment.

## Results

**Figure 2:**
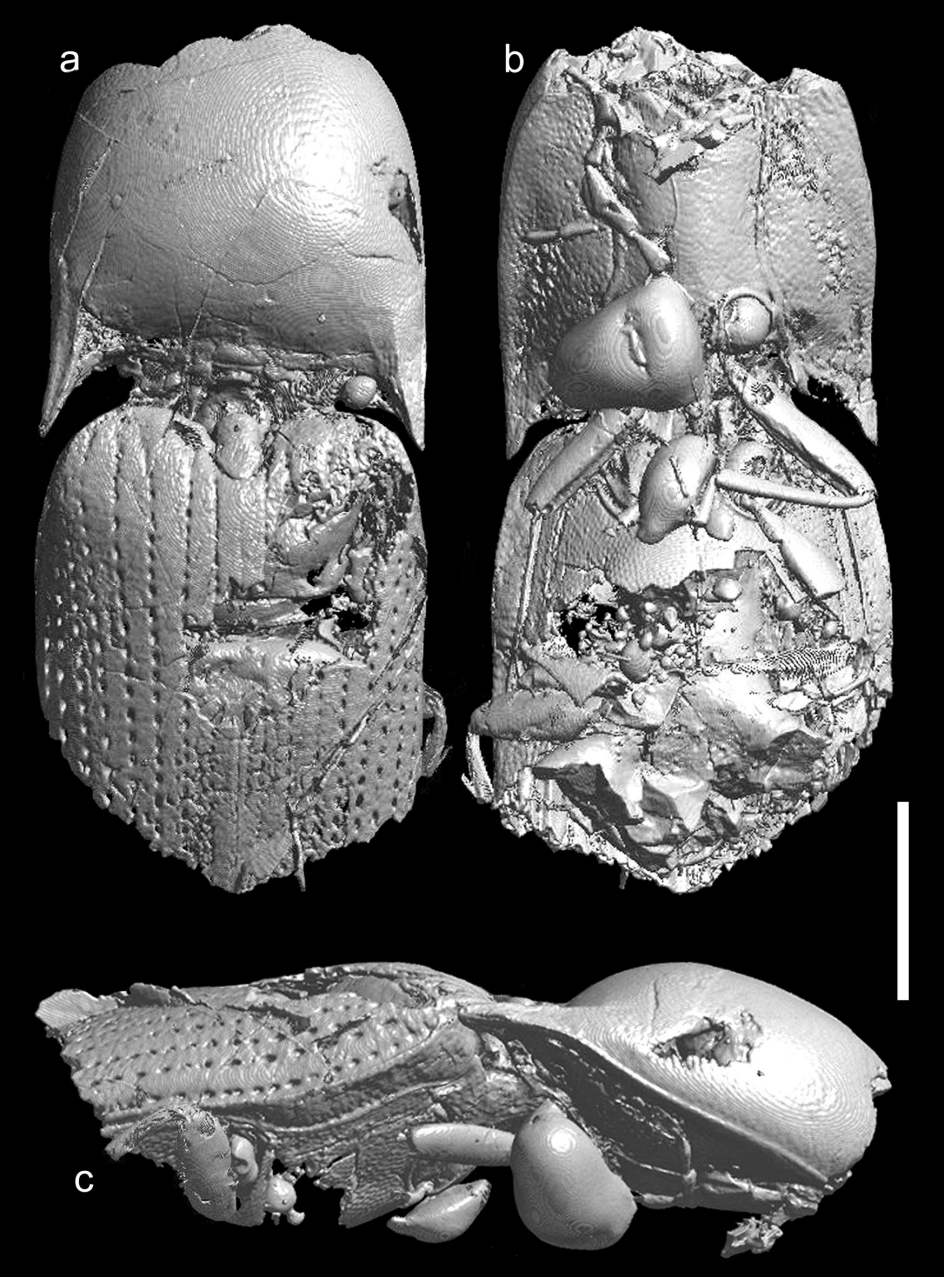
*Ajkaelater merkli* gen. et sp. nov., holotype (MTM PAL 2021.50.1.). (**a**) Dorsal view; (**b**) ventral view; (**c**) right lateral view. Scale bar: 1 mm.

**Figure 3:**
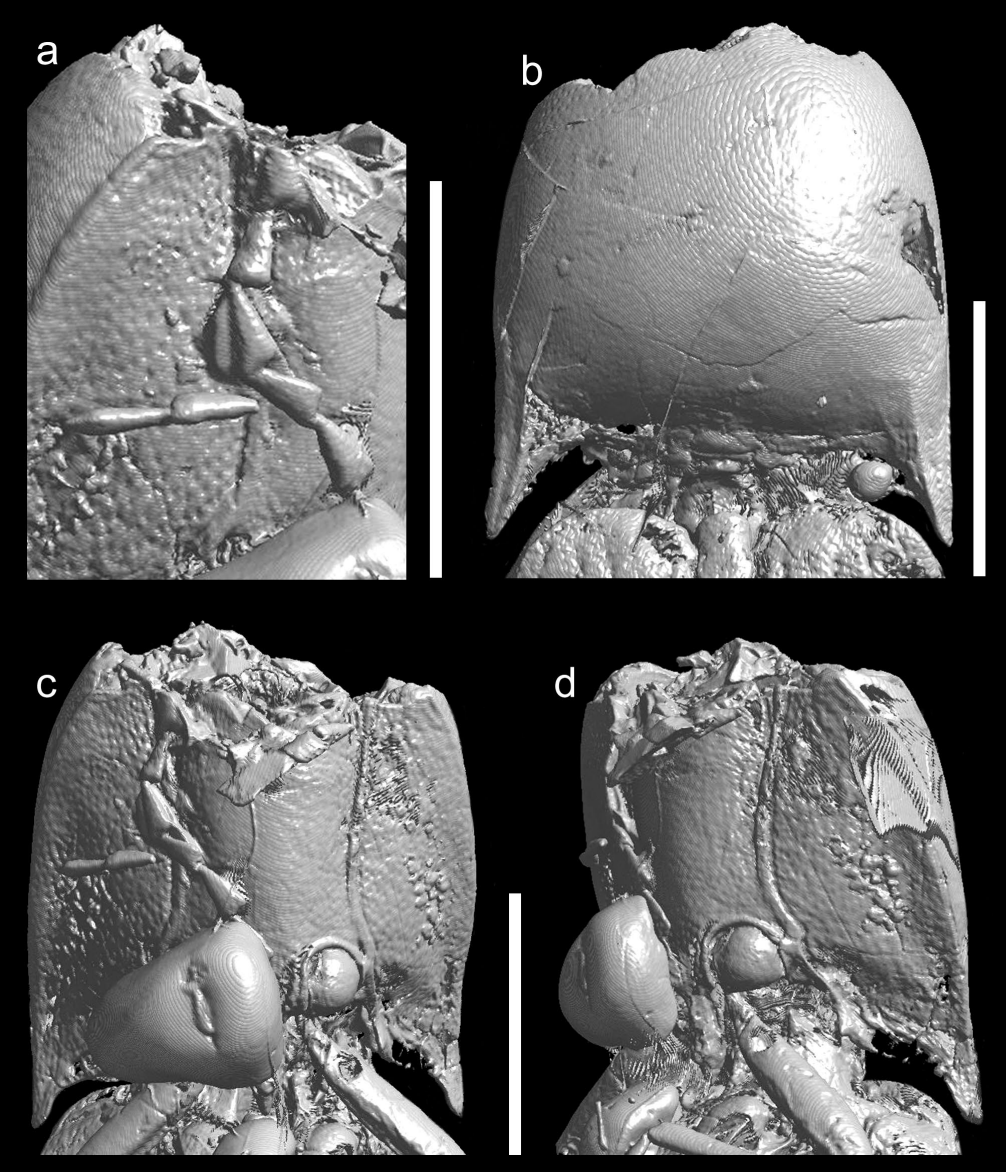
*Ajkaelater merkli* gen. et sp. nov., holotype (MTM PAL 2021.50.1.). (**a**) Preserved antennomeres of the right antenna (in right oblique ventral view); (**b**) prothorax in dorsal view; (**c**) prothorax in ventral view; (**d**) prothorax in left oblique ventral view. Scale bars: 1 mm.

**Figure 4:**
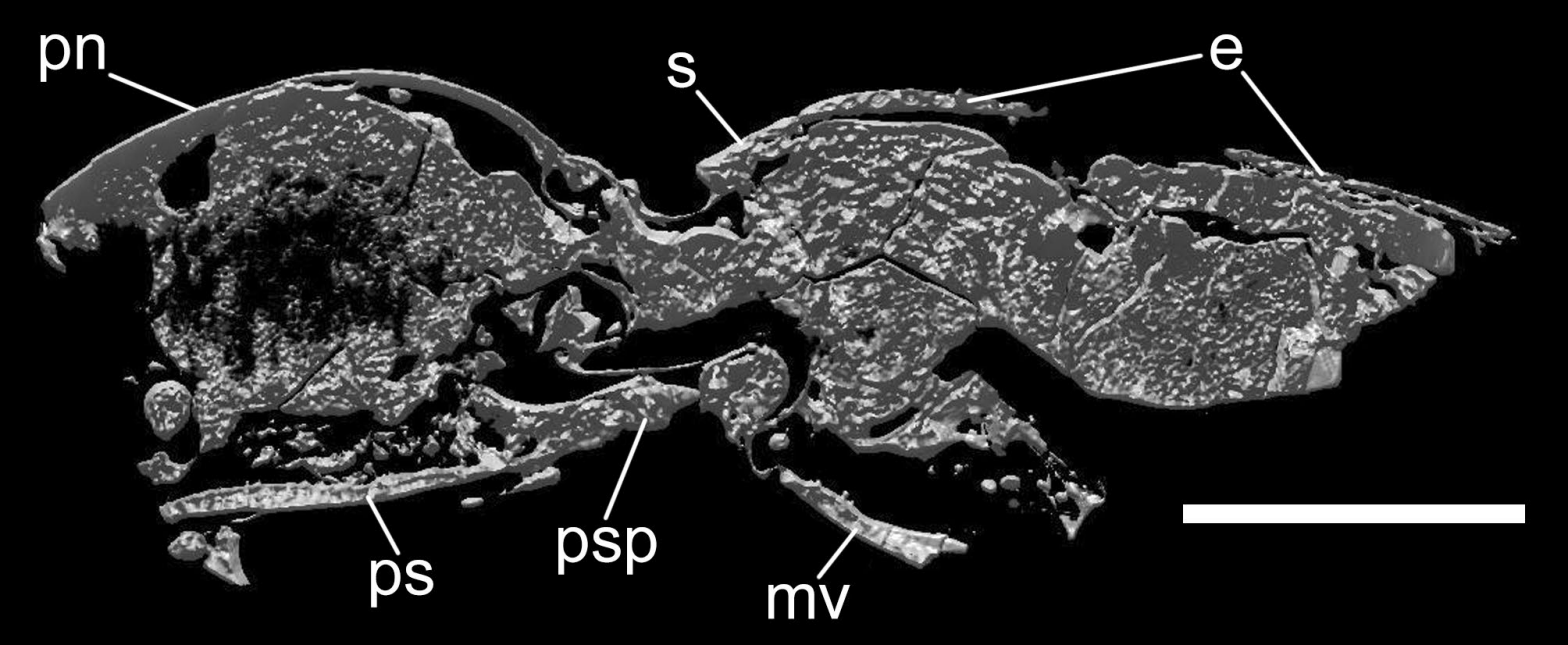
*Ajkaelater merkli* gen. et sp. nov., holotype (MTM PAL 2021.50.1.). Cross section along the saggital axis. *e* elytron, *mv* metaventrite, *s* scutellum, *pn* pronotum, *ps* prosternum, *psp* prosternal process. Scale bar: 1 mm.

**Figure 5:**
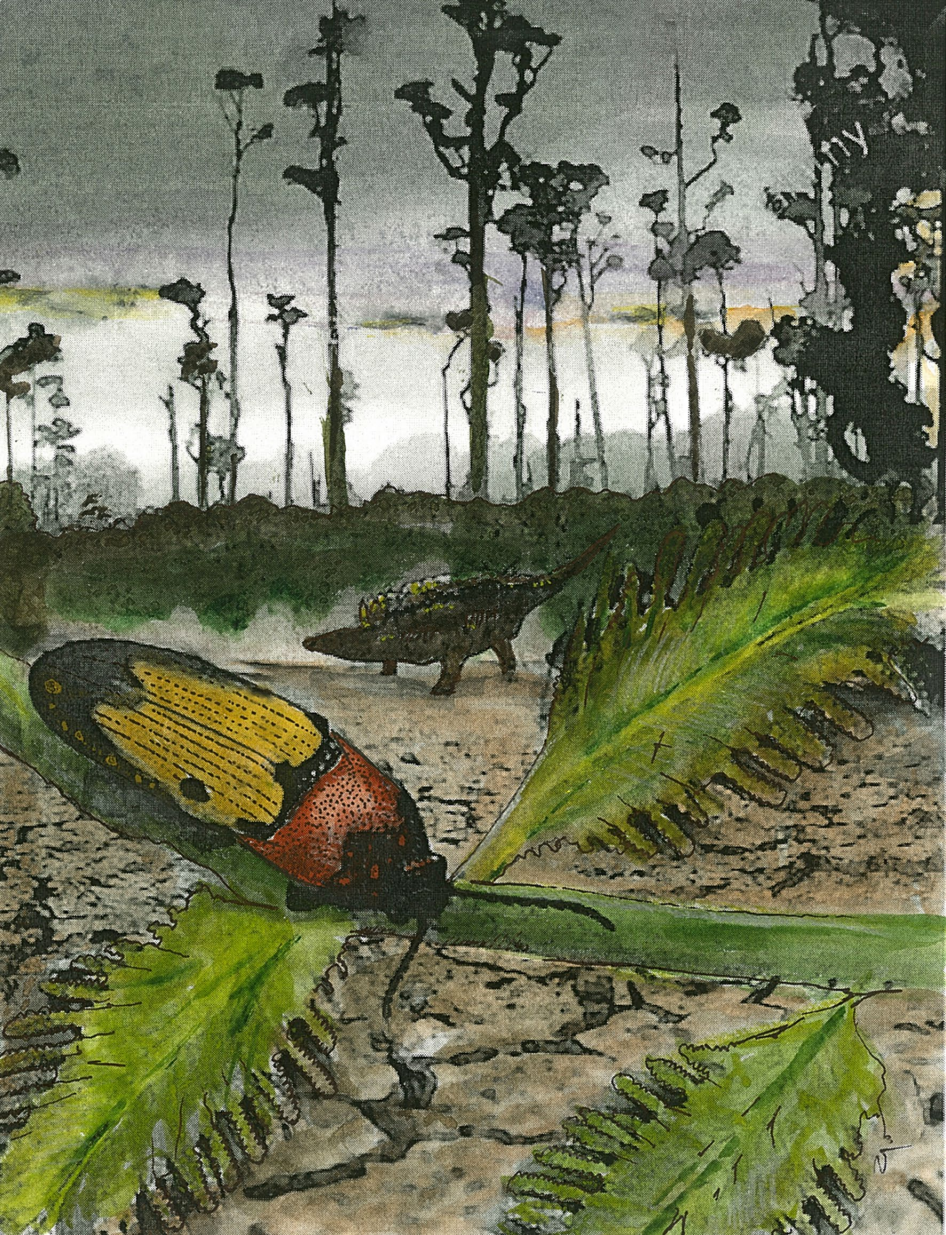
Artistic reconstruction of *Ajkaelater merkli* gen. et sp. nov. in the swampy Ajka coal area in the Santonian western Hungary, with *Hungarosaurus* Ősi, 2005 in the background.

We describe here *Ajkaelater merkli* gen. et sp. nov. (Figs. 2, 3, 4, 5, Supplementary File 1) from Santonian deposits of Ajka based on an incomplete specimen embedded in amber. Based on its habitus and morphology of prothorax, we place it into the click-beetle subfamily Elaterinae, without a tribal assignment (see “Discussion” section).

***Systematic paleontology***

Family Elateridae Leach, 1815

Subfamily Elaterinae Leach, 1815

Tribe *Incertae sedis*

***Ajkaelater*** **gen. nov.**

(Figs. 2, 3, 4, 5, Supplementary File 1)

urn:lsid:zoobank.org:act:F3771C8D-2247-4967-A588-B5505E41AB97

### Type species

*Ajkaelater merkli* gen. et sp. nov.; by present designation.

### Diagnosis

Adult. Body small, oblong-ovate, presumably about 3.25 times as long as wide. Antenna slightly serrate, presumed antennomeres VI–X about twice as long as wide. Pronotum slightly wider than long when measured along midline, widest at posterior angles; lateral sides weakly rounded; posterior angles sharp, slightly divergent; sublateral carina distinct, short; lateral carina distinct, complete; both lateral and sublateral carinae divergent anterad in lateral view. Pronotosternal sutures slightly convergent posterad, then distinctly curved near procoxal cavities. Prosternum elongate and rather narrow, including prosternal process about twice as long as wide, widest near procoxal cavities; prosternal process relatively long, subparallel-sided, abruptly narrowed before apex in lateral view so that apex is on different plane than rest of prosternum. Scutellar shield longer than wide, with anterior margin slightly rounded and steeply declivous. Mesoventral cavity with sides subparallel-sided for greater part of their length, narrowing slightly between mesocoxae. Elytra oblong-ovate, slightly wider than prothorax, sides slightly rounded; each elytron with distinct impressed striae formed by lines of large punctures. For more details, see the description of *A. merkli* gen. et sp. nov. below, Figs. 2, 3, 4, 5, and Supplementary File 1.

### Etymology

Derived from the words “Ajka” (referring to the city of Ajka, the geographic origin of the fossil) and “*Elater*” (a genus name in Elateridae). Gender: masculine.

### Composition and distribution

Only *A. merkli* gen. et sp. nov. (Santonian of Hungary).

***Ajkaelater merkli*** **gen. et sp. nov.**

(Figs. 2, 3, 4, 5, Supplementary File 1)

urn:lsid:zoobank.org:act:15B434D2-B294-4021-B39F-3AE53A846464

### Type material

Holotype, adult, sex unknown, inventory number MTM PAL 2021.50.1. (MTM). A beetle fragment is included in a dark reddish amber piece with dimensions of approximately 4.4 × 3.8 × 1.1 mm (Fig. 1c). There are no visible syninclusions in the amber stone.

Type horizon and age. Ajka Coal Formation, unknown shaft of the Ajka-Csingervölgy coal minery. Based on palynological and nannoplankton investigations, the age of the formation is dated as Upper Cretaceous (Santonian; 86.3–83.6 Ma)^22,29,43,44^.

Type locality. Ajka-Csingervölgy [Ajka-Csinger valley], approximately 1 km SE of the city Ajka, Bakony Mountains, Hungary (Fig. 1a).

### Description

Body (Figs. 2, 3, 5) about 6.50 mm long (rough guess), 2.00 mm wide (measured at humeri), oblong-ovate, subparallel-sided, slightly convex. Head missing, only parts of right antenna preserved, probably with antennomeres VI–IX and X–XI. Preserved antennomeres (Figs. 2b, 3a) weakly serrate, presumed antennomeres VI–X subequal in length, about twice as long as wide, apical antennomere simple, about 1.20 times as long as penultimate antennomere. Pronotum (Figs. 2a, 3b) slightly wider than long when measured along midline (length: 1.75 mm, width: 1.90 mm), widest at posterior angles but only slightly wider there than in about middle. Anterior angles inconspicuous; lateral sides from dorsal view weakly rounded; posterior angles moderately long, sharp, slightly divergent, each medially with distinct sublateral carina running from apex of angle and slightly surpassing posterior margin of pronotum, not subparallel with lateral carina but both carinae divergent anterad in lateral view (Fig. 2c); posterior margin medially with shallow arcuate indentation. Lateral carina (Fig. 2c) distinct, complete, very weakly and gradually sinuate near middle and slightly more sinuate near posterior angle in lateral view. Disc (Figs. 2a, 3b) moderately convex, relatively densely covered with rounded shallow punctures (although punctures not well visible on most micro-CT scans and if visible, then usually only near margins). Hypomeron mostly smooth, with small punctures. Pronotosternal sutures (Figs. 2b, 3c,d) slightly convergent posterad, then distinctly curved near procoxal cavities, their anterior part not well preserved but most probably slightly excavate anteriorly (Fig. 3d). Prosternum (Figs. 2b, 3c,d) elongate, including prosternal process about twice as long as wide, in front of procoxal cavities (i.e., excluding prosternal process) about 1.30 times as long as wide, widest near procoxal cavities; anterior part not well preserved. Prosternal process (Fig. 3d) relatively long, about 0.55 times as long as prosternum in front of procoxal cavities, subparallel-sided, abruptly narrowed before apex in lateral view so that apex is on different plane than rest of prosternum (Fig. 4); apex narrowly rounded. Procoxal cavities subcircular, moderately widely separated by width of prosternal process. Scutellar shield (Figs. 2a, 3b) about 1.50 times as long as wide, anterior margin well defined, angulate, slightly rounded and steeply declivous; lateral sides sinuate, narrowed before middle and widest after middle; apex rounded. Mesoventral cavity deep, with well-defined walls; sides subparallel for greater part of their length, narrowing slightly between mesocoxae. Mesocoxal cavities narrowly separated. Elytra (Fig. 2a) oblong-ovate, slightly wider than prothorax, sides slightly rounded; each elytron with nine distinct impressed striae formed by lines of large subcircular punctures, which are separated by about diameter of a puncture; interstriae flattened, smooth. Epipleuron (Fig. 2c) well developed, wide basally, then gradually narrowed near metacoxae. Fore leg (Fig. 2b) slender, moderately long; profemur about as long as protibia, protarsomere I elongate, about 3 times as long as wide, remaining tarsomeres not preserved; remaining legs incomplete or not preserved. Immature stages unknown.

### Etymology

The specific name “merkli” is a patronym in honor of late Dr. Ottó Merkl (1957–2021), one of the most prominent Hungarian entomologists, the World expert in darkling beetles (Tenebrionidae), and the long-term curator of the Coleoptera Department of the Hungarian Natural History Museum.

## Discussion

Recent rise of interest in research of amber fossils significantly increased the rate of discoveries of various interesting animal lineages with great importance for the understanding of their origin, evolution, and paleodiversity, as well as of the composition of past ecosystems^4^. The here reported discovery of *Ajkaelater merkli* gen. et sp. nov. in the Upper Cretaceous Hungarian amber is of great importance for several reasons.

It is not only the first formally described beetle from ajkaite, but also the first fossil beetle described from the present-day Hungary. So far, some yet undescribed fossil beetles were found in the Miocene and Pliocene deposits of Hungary. The representatives of Dytiscidae and Staphylinidae were reported from the Late Miocene (Sarmatian) of the Tokaj Mountains^45^. Dystiscidae, Carabidae Cerambycidae, Cantharidae, Chrysomelidae, and various Curculionoidea were identified in the Pliocene alginite deposits of Pula in Bakony Mountains^46,47^. Additionally, Krzemiński et al.^46^ reported Carabidae, Hydrophilidae, Silphidae, Chrysomelidae, Lagriinae, and Apioninae from the Pliocene of Gérce in western Hungary. Numerous further beetle inclusions are also known from ajkaite (personal observations of authors), however, their detailed investigation and potential formal description is a matter of future research.

*Ajkaelater merkli* gen. et sp. nov. also represents the first Mesozoic Elateridae described from present-day mainland Europe, and the first Elateridae reported from Santonian worldwide. Based on the fossil record, Elateridae originated either in Triassic (although those records are dubious) or, more probably, Lower Jurassic^40^, and greatly diversified later in Jurassic^39,40,48^. Mesozoic record of click-beetles is mainly composed of species described from the Asian deposits, e.g., Karabastau Formation of Kazakhstan, Zaza Formation of Russia, Yixian, Shanwang, and Jiulongshan Formations of China^48–51^, and only a few species are reported from the Mesozoic deposits of the United Kingdom^40,52^. Regarding the click-beetle inclusions in Mesozoic ambers, three described and many undescribed species are known from the Burmese amber^40^, and a few undescribed species were reported from the Lower Cretaceous Lebanese^53^ and Spanish ambers^14,15^.

The newly described species superficially resembles representatives of the tribe Oophorini (subfamily Agrypninae), which is a cosmopolitan group of click-beetles including several hundred species classified in about 20 genera^54^. The fossil record of Oophorini is known only from the Eocene of the USA and Europe^55,56^, and no fossil of this group has ever been reported from the Mesozoic^40^. Besides the general appearance, *Ajkaelater* gen. nov. shares with Oophorini sharp posterior pronotal angles with a distinct sublateral carina, and the compact elytra with distinctly impressed striae formed by lines of large punctures (Figs. 2, 3). However, these characters can be found in some other click-beetle groups as well, and the most important diagnostic characters which define Oophorini, like the shapes of head and tarsi, the hind wing venation, and the claws^35,57^, cannot be observed as these structures are not preserved in the fossil. What is more, *Ajkaelater* gen. nov. significantly differs from Oophorini in some taxonomically important diagnostic characters, i.e., the shape of prosternum, which is sinuate at sides, with pronotosternal sutures bent inwards (prosternum usually gradually narrowed towards the procoxal cavities in Oophorini, with pronotosternal sutures almost straight), the shape of prosternal process, which is rather stout and notably broadened near procoxae (prosternal process usually rather slender and only slightly widened near procoxae in Oophorini), and the absence of deep incisure at the base of hypomeron (incisure present in Oophorini). This combination of characters is typical for subfamily Elaterinae. Based on its habitus, shapes of antennae, pronotum and elytra, and the absence of incisures in pronotal base, *Ajkaelater* gen. nov. differs from most tribes of Elaterinae, and could be compared only with some Ampedini, Megapenthini, and especially Physorhinini, which sometimes superficially strongly resemble some Oophorini. However, since *Ajkaelater* gen. nov. lacks important structures for the tribal assignment, such as head, complete ventral mesothorax, metacoxal plates, and tarsi, we tentatively place this genus in Elaterinae as *Incertae sedis*. Fossil Elaterinae are known mainly from various Eocene and Miocene deposits^40^, and only a single species was described from the Mesozoic, i.e., *Elater burmitinus* Cockerell, 1917 from the Cretaceous Burmese amber^41^. The discrepancy between the low number of described species from the Mesozoic and the fact that Elaterinae belong to early branches of the Elateridae tree-of-life^38^ can be explained by several factors, including the hard-to-observe subfamilial diagnostic characters, the misplacement of some earlier described taxa, and the general underexamination of click-beetle fossil record^39^. Indeed, Elaterinae are relatively common in Burmese amber (R. Kundrata, personal observation). Our discovery of *Ajkaelater* gen. nov. in the Santonian European deposit further supports the hypothesis that Elaterinae were widely distributed and diversified in Eurasia already in the Cretaceous.

Larvae of extant Elaterinae are very often saproxylic, associated with rotten, decaying wood, but some are also soil dwellers or live in leaf-litter or mosses^35,58–60^. Taking this into account, we suggest that the Ajka Coal Formation paleoenvironment during the Santonian age of the Upper Cretaceous fully matched the assumed ecological needs of *Ajkaelater merkli* gen. et sp. nov. The depositional area of the Ajka Coal Formation was a forested swampy and lacustrine complex ecosystem with presence of both angiosperm and gymnosperm trees^61^, probably dominated by the deciduous trees of *Normapolles* group^62^. Hence, we suggest that the larvae of *Ajkaelater merkli* gen. et sp. nov. were either saproxylic, living in tree trunks or underneath the bark, or occupied a habitat with rich soil in the vicinity of swamps and lakes where the Ajka coal was formed (Fig. 5).

The discovery of *Ajkaelater merkli* gen. et sp. nov. not only significantly contributes to a better understanding of the palaeodiversity and evolution of the click-beetle subfamily Elaterinae in the Cretaceous Europe but also sheds further light on the yet poorly known paleoecosystem and fauna of the Santonian Ajka Coal Formation in present-day western Hungary.

## Material and methods

The ajkaite amber piece, contaning the here described elaterid beetle inclusion (Fig. 1c), was part of the probably largest ajkaite amber piece known to date (approximately 11 × 8 × 3 cm) (see Fig. 2a in Szabó et al.^34^), which was donated to the Hungarian Natural History Museum by late Károly Kozma, the former chief geologist of the Ajka Coal mines. Since it was too large, dark and opaque for classic light microscopic studies, the stone was broken into numerous smaller pieces, some of which contain various representatives of Arachnida, Diptera, Hymenoptera, other Coleoptera, and numerous pieces of plant debris.

The ajkaite sample was scanned using a Bruker Skyscan 2211 nano-CT cone-beam scanner (Skyscan, Bruker, Belgium) at the University of Szeged, Hungary with X-ray source settings of 110 kV source voltage, 700 µA source current and 350 ms exposure time in micro-focus mode using 11 Mp active pixels CCD detector at 2 µm voxel resolution. The 1939 X-ray projections were collected through a 180° rotation of the sample with 0.1° angular step size in around 4.5 h. The acquired images were reconstructed by volumetric NRecon Reconstruction Software (Skyscan, Bruker, Belgium), which uses a modified Feldkamp algorithm. Artifacts which usually occur during reconstruction, such as ring artifact and beam hardening artifacts, were corrected. The 3D model was created using CTVox 3D Micro-CT Volume Rendering software (Skyscan, Bruker, Belgium). Figure 1c was taken with a QImaging MP5.0 digital microscope camera under a Nikon LV 100 polarized light microscope, and processed with Image Pro Insight 8.0 software.

Measurements of the beetle inclusion were taken using the free version of ImageJ 1.48v^63^. In Fig. 1, the map showing location of Ajka coal beds was modified after Császár and Góczán^19^, and the simplified geological section of the Ajka Subbasin was modified after Kozma^64^. The holotype is housed in the collection of the Department of Paleontology and Geology of the Hungarian Natural History Museum in Budapest, Hungary (MTM). The ZooBank LSID number for this publication is: urn:lsid:zoobank.org:pub:BAF89C8F-069E-44FA-A0CE-19A768B0E935.

## Supplementary Information

Supplementary Information.

## Data Availability

All data needed to evaluate the conclusions in the paper are present in the paper, and the video of 3D volume rendering is deposited in Zenodo (see Supplementary File 1; 10.5281/zenodo.5563453).
