# Supplementary material for: The first mainland European Mesozoic click-beetle (Coleoptera: Elateridae) revealed by X-ray micro-computed tomography scanning of an Upper Cretaceous amber from Hungary
Source: Sci Rep. 2022 Jan 7;12:24. doi: 10.1038/s41598-021-03573-5 (PMC8742027; doi:10.1038/s41598-021-03573-5)
Supplement: Supplementary file 1 — Supplementary Information. [file 41598_2021_3573_MOESM1_ESM.pdf]

Supplementary File 1

The first mainland European Mesozoic click-beetle (Coleoptera: Elateridae) revealed by x-ray micro-computed tomography scanning of an Upper Cretaceous amber from Hungary

Márton Szabó[1,2,#], Robin Kundrata[3,#,*], Johana Hoffmannova[3], Tamás Németh[4], Emese Bodor[5,2], Imre Szenti[6], Alexander S. Prosvirov[7], Ákos Kukovecz[6], and Attila Ősi[2,1]

[1]Hungarian Natural History Museum, Department of Paleontology and Geology, Ludovika tér 2, Budapest 1083, Hungary

[2]ELTE Eötvös Loránd University, Institute of Geography and Earth Sciences, Department of Palaeontology, Pázmány Péter sétány 1/C, Budapest 1117, Hungary

[3]Department of Zoology, Faculty of Science, Palacky University, 17. listopadu 50, 771 46, Olomouc, Czech Republic

[4]Department of Zoology and Ecology, Hungarian University of Agriculture and Life Science, 1. Páter K. str., H-2100 Gödöllő, Hungary

[5]Institute for Geological and Geochemical Research, Research Centre for Astronomy and Earth Sciences, Eötvös Loránd Research Network, 1112 Budaörsi street 45, Budapest, Hungary

[6]University of Szeged, Interdisciplinary Centre of Excellence, Department of Applied and Environmental Chemistry, Rerrich Béla tér 1., 6720, Szeged, Hungary

[7]Department of Entomology, Faculty of Biology, Moscow State University, Leninskie gory 1/12, 119234, Moscow, Russia

[#] These authors contributed equally to this work.

*corresponding author; e-mail: robin.kundrata@upol.cz

Video of X-ray microtomography 3D volume rendering of *Ajkaelater merkli* gen. et sp. nov. from Late Cretaceous ajkaite, holotype

- deposited in Zenodo: https://doi.org/10.5281/zenodo.5563453
